# Sex Differences in Cognitive Decline in Subjects with High Likelihood of Mild Cognitive Impairment due to Alzheimer’s disease

**DOI:** 10.1038/s41598-018-25377-w

**Published:** 2018-05-10

**Authors:** Dongwha Sohn, Katie Shpanskaya, Joseph E. Lucas, Jeffrey R. Petrella, Andrew J. Saykin, Rudolph E. Tanzi, Nagiza F. Samatova, P. Murali Doraiswamy

**Affiliations:** 1 North Carolina State University, Department of Computer Science, Raleigh, NC 27695 USA; 2 Oak Ridge National Laboratory, Computer Science and Mathematics Division, Oak Ridge, TN 37831 USA; 3 Stanford University School of Medicine, Department of Radiology, Stanford, CA 94025 USA; 4 Duke University, Department of Statistical Science, Durham, NC 27708 USA; 5 Duke University Medical Center, Department of Radiology, Durham, NC 27710 USA; 6 Indiana University School of Medicine, Indiana Alzheimer Disease Center and the Center for Neuroimaging, Department of Radiology and Imaging Sciences, Indianapolis, IN 46202 USA; 7 Massachusetts General Hospital and Harvard Medical School, Genetics and Aging Research Unit and Department of Neurology, Stanford, CA 02129 USA; 8 Duke University Health System, Neurocognitive Disorders Program, Department of Psychiatry and the Duke Institute for Brain Sciences, Durham, NC 27710 USA

## Abstract

Sex differences in Alzheimer’s disease (AD) biology and progression are not yet fully characterized. The goal of this study is to examine the effect of sex on cognitive progression in subjects with high likelihood of mild cognitive impairment (MCI) due to Alzheimer’s and followed up to 10 years in the Alzheimer’s Disease Neuroimaging Initiative (ADNI). Cerebrospinal fluid total-tau and amyloid-beta (Aβ42) ratio values were used to sub-classify 559 MCI subjects (216 females, 343 males) as having “high” or “low” likelihood for MCI due to Alzheimer’s. Data were analyzed using mixed-effects models incorporating all follow-ups. The worsening from baseline in Alzheimer’s Disease Assessment Scale-Cognitive score (mean, SD) (9 ± 12) in subjects with high likelihood of MCI due to Alzheimer’s was markedly greater than that in subjects with low likelihood (1 ± 6, *p* < 0.0001). Among MCI due to AD subjects, the mean worsening in cognitive score was significantly greater in females (11.58 ± 14) than in males (6.87 ± 11, *p* = 0.006). Our findings highlight the need to further investigate these findings in other populations and develop sex specific timelines for Alzheimer’s disease progression.

## Introduction

Understanding the role of sex in health and disease is a cornerstone of personalized medicine^1,2^. The high failure rate of clinical drug trials in Alzheimer’s disease (AD) over the past decade^3^ has increased the urgency to better dissect the heterogeneity of AD^4^ in order to facilitate more personalized therapies. Females have been noted to be at the epicenter of the AD epidemic due to the fact that they account for roughly two-thirds of AD patients in the US and also the majority of caregivers^1,5,6^. However, despite substantial research investment in AD over decades, the biological role of sex in the neurodegenerative process has been relatively understudied. Laboratory research into AD mechanisms is largely done on male rodents - mirroring the sex bias that exists in many areas of biomedical research where findings from male animals are viewed as generalizable to humans of both sexes^6^.

The higher prevalence of AD in females was largely assumed to be due to their longer life spans compared to men but recent studies are beginning to paint a more complex picture^1,5–23^. In addition to lifespan differences, there are also well known sex differences in other possible AD risk factors such as in genetics, sex hormone changes in midlife, cognitive reserve, and age of onset of comorbid cardio metabolic diseases (reviewed in^1,5,6^) whose interactive effects remain poorly studied. Emerging evidence suggests that female sex may be linked to a greater effect of apolipoprotein ε4 allele (*APOE* ε4) on amyloid pathology and dementia risk as well as a faster rate of cognitive decline after onset of mild cognitive impairment (MCI) or AD^10–17^. In contrast, other studies note that men may have faster verbal memory decline in normal aging^17^, an earlier onset of cardiovascular disease, greater risk for cerebral micro-hemorrhage^10^ and a higher risk for incident MCI^18^. Initial studies of pathological (e.g. beta-amyloid and tau measurements) and neuronal loss (e.g. hippocampal volumetric imaging) biomarkers has also suggested there may be sex differences in the evolution of AD pathophysiology^1,13,20–23^, reviewed in^1^ and^6^. These findings, while preliminary, raise the possibility of multiple points of interaction between sex and AD progression.

The Alzheimer’s Disease Neuroimaging Initiative (ADNI), a multicenter, prospective, naturalistic study (www.adni-info.org), conducted at sites in the US and Canada, has provided new insights into the timeline of evolution of AD biomarkers^24–26^. New NIA-AA recommendations for defining “MCI due to AD – high likelihood”^27^, which require positive pathological (molecular imaging or spinal fluid tests of beta-amyloid and tau) and/or neuronal loss (structural MR imaging) biomarkers in addition to clinical criteria, were, in part, based on MCI data from ADNI. However, these data have not yet been fully examined to study potential sex differences in the progression of subjects with MCI due to AD.

The aims of this study were to examine sex differences in the longitudinal cognitive progression of subjects with high likelihood of MCI due to AD.

## Materials and Methods

### Study Design

The institutional review board at Duke University Health System and at each site reviewed and approved all ADNI protocols. Prior to data collection, all subjects and their legal representatives, when appropriate, gave written informed consent.

Data used in the preparation of this article were obtained from the Alzheimer’s Disease Neuroimaging Initiative (ADNI) (adni.loni.ucla.edu). ADNI was launched in 2003 as a large-scale public-private partnership with a primary goal to investigate whether the integration of clinical assessments, serial imaging studies, and other biological markers can be used to discover early signs of Alzheimer’s Disease (AD) progression. ADNI (ClinicalTrials.gov identifier: NCT00106899) involved over 60 sites across the United States and Canada. ADNI-1 recruited approximately 400 MCI subjects and followed them up to 5 years. These subjects could then choose to continue in ADNI-2 and hence had total follow up of up to 10 years. ADNI-2 recruited approximately 150 new MCI subjects and followed them up to 5 years. Details of protocols and methods can be found in the procedures manual [www.adni-info.org].

### Subjects

Subjects with late MCI enrolled in ADNI-1 and ADNI-2 were eligible for inclusion in this study and were pooled for analyses. All late MCI subjects were between the ages of 55 and 90, had subjective memory complaint, objective memory deficit documented by the Wechsler Memory Scale Logical Memory II, and a Clinical Dementia Rating (CDR) Global of 0.5, did not meet criteria for dementia and had a Geriatric Depression Scale score of less than 6. The diagnostic criteria for late MCI were identical between ADNI-1 and ADNI-2 (http://adni.loni.usc.edu/methods/documents). All subjects met criteria for late MCI. All ADNI-1 and ADNI-2 MCI subjects with at least one post-baseline visit data were eligible for inclusion. In addition to demographic data, for subject inclusion, data for all the following parameters were required: Alzheimer’s Disease Assessment Scale-Cognitive subscale 11 item (ADAS-Cog11) for at least two different time points, *APOE* ε4 genotyping results, and biomarker data. The term “baseline” is used to indicate data collected first at either screening or baseline. The definition of the subset of subjects with a high likelihood of “MCI due to AD” is described under Cerebrospinal fluid (CSF) methods.

### Demographic and Clinical Variables

Demographic variables included were age, sex, education level. Cognitive variables included the ADAS-Cog11 and Mini Mental State Examination (MMSE) [http://www.adni-info.org/].

### *APOE* ε4 Genotyping

*APOE* ε4 allele genotyping of all subjects was completed using DNA extracted from peripheral blood cells as detailed previously^28^.

### MRI Hippocampal Volume Measures

Hippocampal volumes for each subject at baseline were extracted from structural MRI brain scans acquired using a standardized protocol and an automated pipeline using FreeSurfer software (https://surfer.nmr.mgh.harvard.edu/)^25,29^. For this report, only baseline total (right plus left) hippocampal volumes (mm^3^) were used^25^.

### Cerebrospinal fluid (CSF) Assay

Baseline CSF total tau (t-tau), phosphorylated tau_181P_ (p-tau), and amyloid-beta_1–42_ (Aβ42) were analyzed by the ADNI Biomarker Core Laboratory at the University of Pennsylvania Medical Center using the multiplex xMAP Luminex platform (Luminex Corp) with Innogenetics (INNO-BIA AlzBio3, for research use–only reagents) immunoassay kit–based reagents (www.adni-info.org). CSF data was available for approximately one-half of ADNI-1 subjects and most of ADNI-2 subjects. Based on t-tau/Aβ42 ratio values, MCI subjects were sub-classified as having “high” (>0.395 cut-off) or “low” (<0.394 cut-off) likelihood of meeting criteria for MCI due to Alzheimer’s^26^.

### Longitudinal Cognitive tests

MCI subjects were monitored in both ADNI-1 and ADNI-2 at 12 month intervals for up to 5 years. In addition, ADNI-1 MCI subjects could be followed for an additional 5 years if they chose to continue into ADNI-2 thus had a maximum possible follow up of 10 years. At each annual follow visit, subjects underwent cognitive assessments [http://www.adni-info.org/].

### Statistical Analysis

We pooled MCI subjects from ADNI-1 and ADNI-2 studies. Sex-differences in baseline demographic and cognitive variables were tested using either analysis of variance (ANOVA) or analysis of covariance (ANCOVA).

Next we ran three mixed-effect models to examine the effect of sex on change from baseline in ADAS-Cog11. The first model adjusted for age, education, baseline ADAS-Cog11, and *APOE* ε4 allele status as follows:

$$\begin{array}{rcl}\text{ADAS} \mbox{-} \text{Cog}{11}_{j}(t) & = & \mu +{b}_{j}+{\alpha }_{A+}APOE\,{\rm{\varepsilon }}{4}_{A+j}+{{\rm{\alpha }}}_{A++}APOE\,{\rm{\varepsilon }}{4}_{A++j}\\  &  & +\,{{\rm{\alpha }}}_{Age}{{\rm{Age}}}_{j}+{{\rm{\alpha }}}_{Educ}{{\rm{Educ}}}_{j}\\  &  & +\,{{\rm{\alpha }}}_{baselineADAS \mbox{-} Cog11}\,\text{baselineADAS} \mbox{-} \text{Cog}{11}_{j}\\  &  & +\,{{\rm{\alpha }}}_{F}{{\rm{Sex}}}_{Fj}+{\beta }_{A+}APOE\,{\rm{\varepsilon }}{4}_{A+j}t\\  &  & +\,{\beta }_{A++}APOE\,{\rm{\varepsilon }}{4}_{A++j}t+{\beta }_{Age}{{\rm{Age}}}_{j}t+{\beta }_{Educ}{{\rm{Educ}}}_{j}t\\  &  & +\,{\beta }_{baselineADAS \mbox{-} Cog11}\,{\rm{baseline}}\,\text{ADAS} \mbox{-} Cog{11}_{j}t\\  &  & +\,{\beta }_{F}{{\rm{Sex}}}_{Fj}t+{\gamma }_{F}{{\rm{Sex}}}_{Fj}{t}^{2}+{\gamma }_{A+}APOE\,{\rm{\varepsilon }}{4}_{A+j}{t}^{2}\\  &  & +\,{\gamma }_{A++}APOE\,\varepsilon {4}_{A++j}{t}^{2}+{\beta }_{0}t+{\gamma }_{0}{t}^{2}+{{\rm{r}}}_{j}t+{{\rm{\varepsilon }}}_{jt}\end{array}$$

In this model, the follow-up time (month) was centered with the median follow-up time and covariates were centered i.e. a 75 years old *APOE* ε4- male with 16 years of education and an ADAS-Cog11 score of 11. We included both random slope and random intercept of each subject in this mixed effect model to account for subject-specific variability in each baseline dependent variable and rate of change, respectively, as reported previously^14^. Square root transformations were used for all dependent variables in all models to obtain approximate normality of estimated error distribution and homoscedasticity (constant variance) of the errors across fitted values of each dependent variable. In the models, *APOE* ε4 status was treated as a categorical variable while age, education, cognitive scores were treated as continuous variables. In the equation, *APOE* ε4 ++ indicates carriers of two *APOE* ε4 alleles (homozygous) while *APOE* ε4 + indicates carriers of one *APOE* ε4 allele (heterozygous). We also ran a second mixed effect model replacing APOE ε4 with biomarkers (baseline t-tau, Aβ42 or hippocampal volume) as covariates. Next, we ran a mixed effects model in subjects with high likelihood of MCI due to AD selected two ways. The first was based on high t-tau/Aβ42 ratio. In this model, *APOE* ε4 status was not included as a term in this last model due to its collinearity with Aβ42. We also ran a mixed effects model in MCI subjects who are *APOE* ε4 positive. The model terms and covariates are all described in the Tables. Not all analyses had the same sample sizes due to missing values or drop outs. All statistical analyses were conducted in the R (www.r-project.org); mixed-effect models for longitudinal analyses were conducted using the nlme package in the R. All methods were performed in accordance with the relevant guidelines and regulations.

## Results

Table 1 summarizes the baseline demographics of the 559 MCI subjects included in this study. Female subjects were younger than male MCI subjects (*p* = 0.002). There was no statistically significant difference in *APOE* ε4 carrier status between males and females.

**Table 1:** Baseline Demographic and Clinical Characteristics by Sex of Subjects.

ADNI MCI	All	Female	Male	p-value
No. subjects	559	216	343	
Age (years)	74.0/7.5	72.8/7.6	74.8/7.4	**0.002**
Education (years)	15.9/2.9	15.4/2.8	16.2/3.0	**0.004**
MMSE	27.2/1.8	27.1/1.8	27.2/1.8	0.492
ADAS-Cog11	11.5/4.6	11.2/4.8	11.7/4.4	0.158
*APOE* ε4 carriers (%)	54	58	52	0.196
MCI due to AD – high likelihood (%)	69.5	73.0	67.3	
Follow-up Duration (months)	43.8/29.6*	42.4/27.3*	44.8/30.9*	0.351
Total hippocampal volume (mm^3^)	5901.1/ 1079.3 (n = 443)	5678.9/1073.5 (n = 168)	6036.9/ 1062.0 (n = 275)	**<0.001**
**“MCI due to AD – high likelihood”**	**All**	**Female**	**Male**	**p-value**
No. subjects	244	100	144	
Age (years)	73.6/7.3	71.3/7.2	75.1/6.9	**<0.001**
Education (years)	16.1/2.8	15.5/2.8	16.5/2.8	**0.006**
MMSE	27.0/1.8	26.9/1.8	27.0/1.9	0.663
ADAS-Cog11	12.6/4.9	12.3/4.9	12.8/4.8	0.511
APOE ε4 carrier (%)	70	76	65	0.098
Follow-up Duration (months)	40.4/24.8	38.7/22.9	39.7/27.0	0.770
Total hippocampal volume (mm^3^)	5773.4/1006.0 (n = 184)	5622.2/1011.4 (n = 77)	5882.2/992.6 (n = 107)	0.084

ANOVA (Analysis of variance) assessed differences in age, education year, follow-up duration and ANCOVA (Analysis of covariance) assessed sex-differences in baseline MMSE and ADAS-Cog11 scores adjusting for age, years of education. Data are expressed as mean/standard deviation, as appropriate. Bold *p*-values are statistically significant. Abbreviations: AD (Alzheimer’s disease), MCI (mild cognitive impairment), ADAS-Cog11 (Alzheimer’s disease assessment scale- cognitive subscale), MMSE (mini- mental state examination), and *APOE* ε4 (apolipoprotein ε4 allele). Follow-up duration is calculated based on ADAS-Cog 11 measurement. CSF t-tau/Aβ42 ratio cut-offs were used to classify subjects as having “high” likelihood of meeting criteria for “MCI due to AD”. See text for details.

### Effect of sex on longitudinal cognitive decline

Mean follow up duration (months) for males (44.8 ± 30.9) did not significantly differ from that of females (42.4 ± 27.3). The mean (±SD) change from baseline in ADAS-Cog11 in females (8.7 ± 12.6) was greater than in males (5.8 ± 10.1, *p* = 0.001). The mean change from baseline in ADAS-Cog11 in female *APOE* ε4 carriers (10 ± 14) and non-carriers (7 ± 10) was greater than in male *APOE* ε4 carriers (8 ± 11) (*p* = 0.04) and non-carriers (3 ± 9) (*p* = 0.003), respectively.

Table 2 depicts the mixed effects model testing for sex and *APOE* ε4 effects on longitudinal change in ADAS-Cog11 in MCI subjects. In this model, sex had a significant effect on ADAS-Cog11 slope (*p* = 0.003) with the cognitive decline being greater in females than males. Baseline cognition, education and *APOE* ε4 status also had a significant effect. Subjects with worse baseline cognition and higher education declined faster. *APOE* ε4 heterozygotes and homozygotes declined faster, and *APOE* ε4 had a significant effect on both slope and curvature of ADAS-Cog11 change (compared to non-carriers). The effect of interaction between sex and *APOE* ε4 on ADAS-Cog11 change was not significant (Supplementary Table 1).

**Table 2:** Effect of Sex and *APOE* ε4 on longitudinal change in ADAS-Cog11 of MCI subjects.

Term	Coefficient	Standard error	t-value	p-value
Intercept	3.544899	0.06782345	52.26658	**<0.001**
Sex effect	0.255545	0.08529762	2.99592	**0.003**
*APOE* ε4 + effect	0.308539	0.08876979	3.47572	**<0.001**
*APOE* ε4 + + effect	0.568898	0.13237472	4.29763	**<0.001**
Baseline rate	0.012660	0.00181201	6.98689	**<0.001**
Baseline curvature	0.000099	0.00001834	5.39852	**<0.001**
Age effect	0.011337	0.00560956	2.02101	**0.044**
Education effect	0.016933	0.01428969	1.18500	0.237
Baseline cognition effect	0.166261	0.00932751	17.82485	**<0.001**
Female effect on slope	0.006905	0.00231191	2.98663	**0.003**
Female effect on curvature	0.000006	0.00002605	0.24503	0.807
*APOE* ε4 + effect on slope	0.010323	0.00238571	4.32693	**<0.001**
*APOE* ε4 + + effect on slope	0.018991	0.00362755	5.23525	**<0.001**
*APOE* ε4 + effect on curvature	0.000071	0.00002589	2.76107	**0.006**
*APOE* ε4 + + effect on curvature	0.000115	0.00004004	2.87349	**0.004**
Education effect on slope	0.000780	0.00038381	2.03293	**0.042**
Age effect on slope	0.000142	0.00015109	0.94080	0.347
Baseline cognition effect on slope	0.001074	0.00025382	4.23056	**<0.001**

Baseline cognition indicates ADAS-Cog 11. Bold *p*-values are statistically significant. Abbreviations: MCI (mild cognitive impairment), ADAS-Cog11 (Alzheimer’s disease assessment scale- cognitive subscale), and *APOE* ε4 (apolipoprotein ε4 allele). In this model, the follow-up time (month) was centered with the median follow-up time (36 months) and covariates were centered i.e. a 75 years old *APOE* ε4- male with 16 years of education and an ADAS-Cog11 of 11. Table depicts that the effect of sex on ADAS-Cog11 change was significant with females declining faster than males. Education and baseline cognition also had significant effects. The intercept is a term to get the correct estimate of the outcome when time = 0. The baseline rate is the reference population rate of change in the outcome starting at time zero, and the baseline curvature is the “acceleration” of that rate of change at time zero. The upper half of the table shows the effect of specific variables on ADAS-Cog11 and the bottom half shows their effects on ADAS-Cog11 slope and curvature.

Table 3 depicts the mixed effects model testing the effect of sex and baseline CSF Aβ42 (as a continuous measure) on longitudinal change in ADAS-Cog11 in MCI subjects. In this model, sex (*p* = 0.027), baseline CSF Aβ42 (*p* < 0.001) had a significant effect on ADAS-Cog11 change. Females and subjects with lower CSF Aβ42 declined faster. Aβ42 also had a significant effect on curvature of ADAS-Cog11 change (*p* < 0.001). The effect of age, education, baseline cognition was not significant. Supplementary Table 2 depicts the effect of sex and baseline CSF t-tau on ADAS-Cog11 change. In this model, the effect of sex was not significant but patients with higher CSF t-tau had greater ADAS-Cog11 worsening (*p* < 0.001). Supplementary Table 3 depicts the effect of sex and baseline total hippocampal volume on ADAS-Cog11 change. Baseline total hippocampal volumes were smaller in females (than males). However, when normalized as a ratio to intracranial volume, they were significantly larger than that of males. In the mixed model of ADAS-Cog11 change, the effect of sex was not significant but the effect of baseline total hippocampal volume was significant (*p* < 0.0001).

**Table 3:** Effect of Sex and CSF Aβ42 on longitudinal change in ADAS-Cog11 of MCI subjects.

Term	Coefficient	Standard error	t-value	p-value
Intercept	3.877795	0.06891898	56.26600	**<0.001**
Sex effect	0.223058	0.10668516	2.09081	**0.037**
Aβ42 effect	−0.007031	0.00100517	−6.99508	**<0.001**
Baseline rate	0.023720	0.00184203	12.87688	**<0.001**
Baseline curvature	0.000177	0.00002078	8.52360	**<0.001**
Age effect	0.002999	0.00680578	0.44068	0.660
Education effect	0.000530	0.01816607	0.02918	0.977
Baseline cognition effect	0.139866	0.01143890	12.22720	**<0.001**
Female effect on slope	0.006420	0.00290663	2.20866	**0.027**
Female effect on curvature	−0.000006	0.00003607	−0.15359	0.878
Baseline Aβ42 effect on slope	−0.000216	0.00002632	−8.21396	**<0.001**
Baseline Aβ42 effect on curvature	−0.000001	0.00000028	−3.52631	**<0.001**
Education effect on slope	0.000301	0.00048418	0.62259	0.534
Age effect on slope	−0.000006	0.00018281	−0.03192	0.975
Baseline cognition effect on slope	0.000378	0.00030937	1.22315	0.221

Baseline cognition indicates ADAS-Cog 11. Bold *p*-values are statistically significant. Abbreviations: MCI (mild cognitive impairment), ADAS-Cog11 (Alzheimer’s disease assessment scale- cognitive subscale), and Aβ42 (amyloid-beta_1–42_). In this model, the follow-up time (month) was centered with the median follow-up time (36 months) and covariates were centered i.e. a 75 years old with 16 years of education, ADAS-Cog11 of 11, and Aβ42 of 147. Table depicts that the effect of sex on ADAS-Cog11 slope was significant with females declining faster than males. Baseline Aβ42 also had a significant effect on both slope and curvature. Age and education did not have significant effects on slope. The intercept is a term to get the correct estimate of the outcome when time = 0. The baseline rate is the reference population rate of change in the outcome starting at time zero, and the baseline curvature is the “acceleration” of that rate of change at time zero. The upper half of the table shows the effect of specific variables on ADAS-Cog11 and the bottom half shows their effects on ADAS-Cog11 slope and curvature.

### Sex differences in Subjects with High Likelihood of MCI due to AD

**Figure 1:**
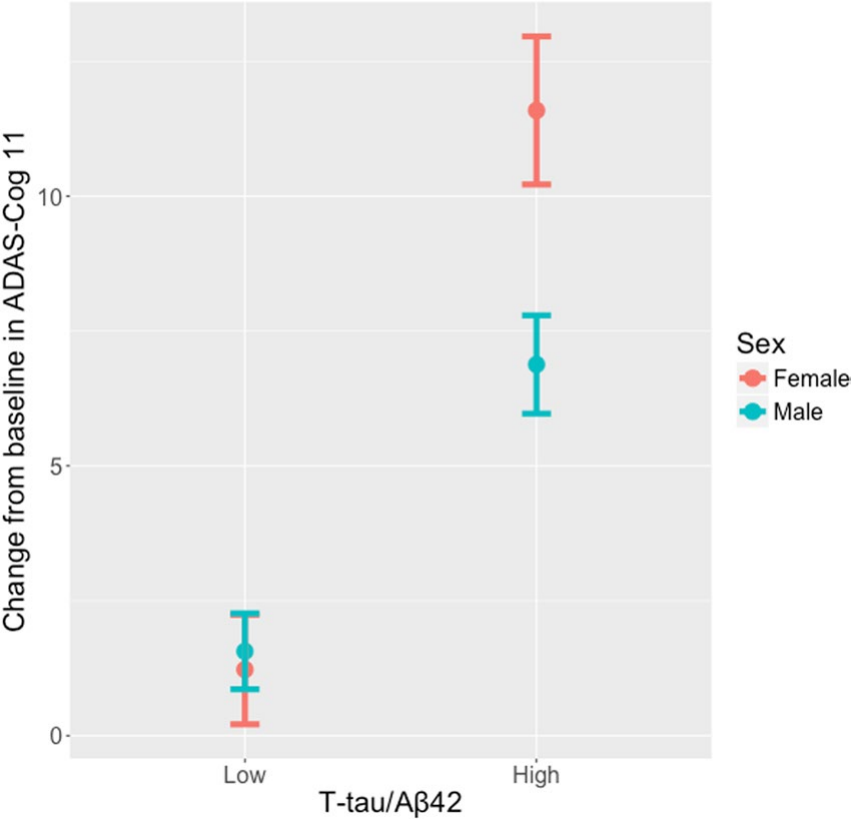
ADAS-Cog11 change in subjects with high or low probability of MCI due to AD. Y-axis depicts the mean (SE) change from baseline in ADAS-Cog11 of MCI subjects by sex. X-axis depicts the grouping by CSF t-tau/Aβ42 ratio into “high” or “low” likelihood of having MCI due to AD. MCI due to AD high probability subjects had greater change than those with low probability. Among subjects with high probability of MCI due to AD, females showed greater change than males. Data comprises pooled MCI subjects from ADNI-1 and ADNI-2.

**Figure 2:**
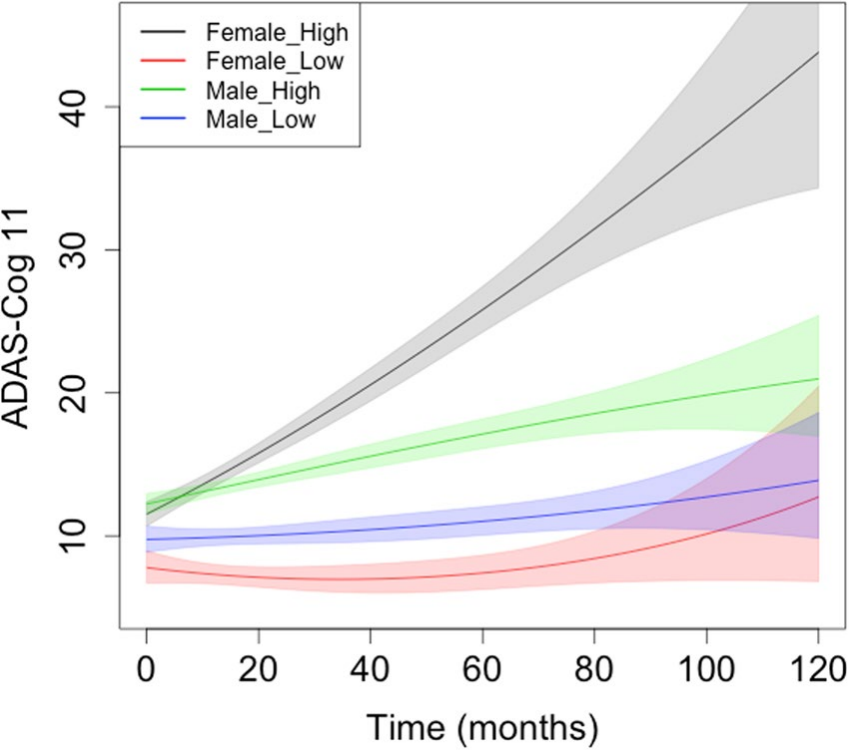
ADAS-Cog11 Slopes in subjects with high or low probability of MCI due to AD. X-axis depicts maximum duration of follow up. Y-axis depicts ADAS-Cog11 scores. MCI subjects have been grouped using CSF t-tau/Aβ42 ratio as having “high” or “low” probability of MCI due to AD. Slopes and confidence intervals are derived from a simple quadratic model (polynomial regression) by sex over time without any other covariates. Data comprises pooled MCI subjects from ADNI-1 and ADNI-2. Female subjects with high probability of MCI due to AD showed greater decline than the other groups.

As described in Methods, we used t-tau/Aβ42 ratio cut-off to identify subjects with “MCI due to AD – high likelihood”. 70% of all MCI subjects, 73% of females and 67% of males met this criterion for MCI due to AD (Table 1). The mean (±SD) change from baseline in ADAS-Cog11 (9 ± 12) in subjects with “MCI due to AD – high likelihood” subjects was significantly greater than that in MCI subjects who did not meet such criteria (1 ± 6, *p* < 0.0001). Among subjects with “MCI due to AD – high likelihood”, the mean worsening in ADAS-Cog11 was significantly greater in females (12 ± 14) than in males (7 ± 11, *p* = 0.006) (Figs 1 and 2). Table 4 depicts the mixed effects model testing the effect of sex on ADAS-Cog11 change in subjects with MCI due to AD – in this model, the effect of sex was significant on ADAS-Cog11 slope (*p* = 0.021). In this model, age, education and baseline cognition did not have a significant effect.

**Table 4:** Effects of Sex on ADAS-Cog11 change in MCI due to AD – high likelihood.

Term	Coefficient	Standard error	t-value	p-value
Intercept	3.964566	0.09028821	43.91011	**<0.001**
Sex effect	0.384134	0.14181187	2.70876	**0.007**
Baseline rate	0.026903	0.00255143	10.54425	**<0.001**
Baseline on curvature	0.000196	0.00002650	7.41382	**<0.001**
Age effect	0.005377	0.00955933	0.56247	0.574
Education effect	0.026654	0.02443509	1.09082	0.277
Baseline cognition effect	0.131962	0.01403214	9.40425	**<0.001**
Female effect on slope	0.009472	0.00410204	2.30907	**0.021**
Female effect on curvature	−0.000003	0.00004764	−0.07276	0.942
Education effect on slope	0.000881	0.00068796	1.28007	0.201
Age effect on slope	−0.000066	0.00026925	−0.24662	0.805
Baseline cognition effect on slope	0.000246	0.00039833	0.61665	0.538

CSF t-tau/Aβ42 ratio was used to identify subjects with MCI due to AD – high likelihood. Baseline cognition indicates ADAS-Cog 11. Bold *p*-values are statistically significant. Abbreviations: MCI (mild cognitive impairment), ADAS-Cog11 (Alzheimer’s disease assessment scale- cognitive subscale). In this model, the follow-up time (month) was centered with the median follow-up time (36 months) and covariates were centered i.e. a 75 years old with 16 years of education and an ADAS-Cog11 of 11. The effect of sex was significant with females declining faster than males. The intercept is a term to get the correct estimate of the outcome when time = 0. The baseline rate is the reference population rate of change in the outcome starting at time zero, and the baseline curvature is the “acceleration” of that rate of change at time zero. The upper half of the table shows the effect of specific variables on ADAS-Cog11 and the bottom half shows their effects on ADAS-Cog11 slope and curvature.

### Sex differences in *APOE* ε4 positive MCI subjects

Table 5 depicts results of a mixed effect model testing for sex differences in ADAS-Cog11 change over time in *APOE* ε4 positive MCI subjects (including both heterozygotes and homozygotes). In this model, sex had a near significant effect on ADAS-Cog11 slope (*p* = 0.05) with the cognitive decline being greater in females than males.

**Table 5:** Effects of Sex on ADAS-Cog11 change in MCI *APOE* ε4 carriers.

Term	Coefficient	Standard error	t-value	p-value
Intercept	3.922808	0.07839959	50.03608	**<0.001**
Sex effect	0.330213	0.12658675	2.60859	**0.010**
Baseline rate	0.025747	0.00213309	12.07038	**<0.001**
Baseline on curvature	0.000199	0.00002031	9.79324	**<0.001**
Age effect	0.017849	0.00888039	2.00998	**0.045**
Education effect	0.034823	0.02094617	1.66248	0.098
Baseline cognition effect	0.155364	0.01336529	11.62443	**<0.001**
Female effect on slope	0.006926	0.00353623	1.95851	**0.050**
Female effect on curvature	−0.000029	0.00003745	−0.78379	0.433
Education effect on slope	0.001089	0.00057509	1.89437	0.058
Age effect on slope	0.000240	0.00024422	0.98105	0.327
Baseline cognition effect on slope	0.001011	0.00037038	2.72871	**0.006**

Baseline cognition indicates ADAS-Cog 11. Bold *p*-values are statistically significant. Abbreviations: MCI (mild cognitive impairment), ADAS-Cog11 (Alzheimer’s disease assessment scale- cognitive subscale). In this model, the follow-up time (month) was centered with the median follow-up time (36 months) and covariates were centered i.e. a 75 years old with 16 years of education and an ADAS-Cog11 of 11. The effect of sex on ADAS-Cog11 slope was near significant. The intercept is a term to get the correct estimate of the outcome when time = 0. The baseline rate is the reference population rate of change in the outcome starting at time zero, and the baseline curvature is the “acceleration” of that rate of change at time zero. The upper half of the table shows the effect of specific variables on ADAS-Cog11 and the bottom half shows their effects on ADAS-Cog11 slope and curvature.

### ADNI-1 versus ADNI-2

Supplementary Figures 1 and 2 depict the mean change from baseline to last observation as well as the slopes (derived from a simple quadratic model) of ADAS-Cog11 change in males and females in ADNI-1 and ADNI-2 separately for subjects with MCI due to AD high probability. Of the overall MCI sample, there were 397 from ADNI-1 and 162 from ADNI-2. The mean follow up of ADNI-1 subjects was 48.2 and for ADNI-2 subjects was 33.2. In both ADNI-1 and ADNI-2, MCI due to AD high probability subjects declined much faster than biomarker negative subjects (Supplementary Figures 1 and 2). Among MCI due to AD subjects, significant longitudinal sex differences were seen in ADNI-1 MCI but not in ADNI-2.

## Discussion

Understanding the potential underpinnings of sex-related differences in the risk for dementia is an important research priority for the field^1–7^. Our study systematically examined sex differences in longitudinal cognitive outcomes using pooled MCI data from two multicenter studies, ADNI-1 and ADNI-2. To our knowledge, this is also the first report to examine sex differences in outcomes of MCI subjects defined using pathological CSF biomarkers to have a high likelihood for MCI due to Alzheimer’s.

Several interesting findings emerged from this study. Our longitudinal cognitive analyses of the pooled dataset found that MCI females showed greater cognitive decline than males. Our study also found that *APOE* ε4 has an effect on both slope and curvature of ADAS-Cog11 decline and with both heterozygotes and homozygotes declining faster than non-carriers. Further among MCI *APOE* ε4 carriers, we found that females declined faster than males. We found no interaction effect between sex and *APOE* ε4 suggesting these variables may potentially have additive but not multiplicative effects. Lastly, we used CSF biomarkers to identify subjects with “high” or “low” probability of having MCI due to AD. Although not a perfect classifier, the tau/Aβ42 ratio cut-off we used has been validated in a clinic-pathological study^25^, used in published studies e.g.^30,31^, and cited in the NIA-AA guideline report on diagnosing MCI due to AD^27^. Approximately 70% of the ADNI MCI sample met these surrogate criteria for “MCI due to AD – high likelihood”. MCI due to AD subjects showed a markedly greater cognitive decline (almost 9-fold) than MCI subjects not meeting these criteria. This further supports the utility of the CSF ratio as a potential prognostic marker for selecting subjects at high risk for decline in clinical trials. Further, among the “MCI due to AD – high likelihood” group, females showed greater cognitive decline than males. This finding extends prior reports of sex differences in MCI^13,14,23^ and to our knowledge is the first study to examine sex differences in subjects with “MCI due to AD – high likelihood”.

There are some strengths and limitations to our study. A major strength of the ADNI study data is that it represents a multicenter biomarker study that recruited subjects from over 60 sites in the US and Canada and performed longitudinal clinical and biomarker assessments using a highly standardized protocol^24^. ADNI results have, in part, formed the basis for entry criteria in many prevention trials and hence ADNI is a highly relevant dataset. Our analyses tried to mimic the emerging new criteria for MCI due to AD – high likelihood using pathological CSF biomarkers. There is as such no definitive binary marker for neuronal loss and hence we relied on pathological markers. The relatively large sample size and long duration of follow up are other strengths of the analyses. There are also some limitations. ADNI subjects were recruited largely at clinic-based research sites for a biomarker study and as such may not reflect milder subjects seen in general practice, especially in primary care settings. While we relied on a pathologically validated^25^ tau/Aβ42 ratio cut-off as a surrogate to identify biomarker positive MCI subjects, there is as yet no perfect *in-vivo* method to identify MCI due to AD. While the diagnostic utility of CSF tau/Aβ42 ratio may differ by setting and laboratory^32^, it still remains useful for identifying a subset of rapid decliners. While baseline sex differences were seen in both studies, longitudinal sex differences were driven primarily by ADNI-1 data and were not significant in ADNI-2. We do not know why, but one possibility may be that ADNI-1 recruited twice as many MCI subjects as ADNI-2 and ADNI-1 subjects could have a 120 month maximum follow up (versus 60-month maximum follow up in ADNI-2). Although entry criteria were the same for late MCI between ADNI-1 and ADNI-2, we cannot rule out the possibility of selection bias since the two studies were done 5 years apart. Differences in comorbid conditions and concomitant medications between ADNI studies may also have contributed. Since the follow up period was roughly the same between males and females, the observed differences are less likely to be due to attrition or survival biases but we cannot rule them out. We studied sex effects on the ADAS-Cog11 as it is frequently used in clinical trials. However, it is not necessarily perfectly balanced across all cognitive domains and there is some evidence that females may show a slightly different profile (as a group) than males on this test. To rule out potential testing bias, it is important to also examine sex differences on other cognitive and functional domains. Hence, these issues must be kept in mind while interpreting the data and our findings must be viewed as initial pending replication in population samples.

Our study does not directly address mechanisms that may underlie potential sex differences in MCI progression. Theories proposed have included a greater potency of the AD risk associated with the *APOE* ε4 allele and the BDNF Met66 allele in females, differences in sex hormones, smaller head size, lower cognitive reserve, as well as the possibility of differential expression of a variety of genes (reviewed in^1,5–23^). The disappearance of sex effects after co-varying for total hippocampal volumes and total tau levels suggest these may be somehow related. However, because AD involves multiple biochemical alterations, systems biology approaches to examine sex differences at a network and pathway level are warranted and may yield deeper insights^28,33,34^. In summary, our findings of sex differences in both baseline biomarkers and cognitive progression in biomarker defined MCI subjects highlight the need to further investigate sex specific biomarker evolution and disease progression in AD.

## Electronic supplementary material

Supplementary Information
