# Supplementary material for: Sex Differences in Cognitive Decline in Subjects with High Likelihood of Mild Cognitive Impairment due to Alzheimer’s disease
Source: Sci Rep. 2018 May 10;8:7490. doi: 10.1038/s41598-018-25377-w (PMC5945611; doi:10.1038/s41598-018-25377-w)
Supplement: Supplementary file 1 — Supplementary Information [file 41598_2018_25377_MOESM1_ESM.docx]

**Sex Differences in Cognitive Decline in Subjects with High Likelihood of**

**Mild Cognitive Impairment due to Alzheimer’s disease**

Dongwha Sohn^1,2^, Katie Shpanskaya^3^, Joseph E. Lucas^4^, Jeffrey R. Petrella^5^, Andrew J. Saykin^6^, Rudolph E. Tanzi ^7^, Nagiza F. Samatova^1,2^, P. Murali Doraiswamy^8^

**Supplementary materials**

Supplementary figure 1. ADAS-Cog11 change from baseline by sex in ADNI 1 (left) and ADNI2 (right) studies.

ADNI-1 ADNI-2

The x-axis depicts the use of cerebrospinal fluid (CSF) pathological markers to group subjects with “high” or “low” probability of MCI due to AD (see text for details). Y-axis depicts mean (SE) change from baseline to last observation. In both ADNI-1 and ADNI-2, subjects with high probability declined faster than those with low probability. In ADNI-1 sex differences are significant in the MCI due to AD high probability group with females showing greater change than males. The effect of sex is not significant in ADNI-2.

Supplementary figure 2. Slopes of ADAS-Cog11 change in “MCI due to AD – high likelihood” in ADNI 1 (left) and ADNI2 (right) study


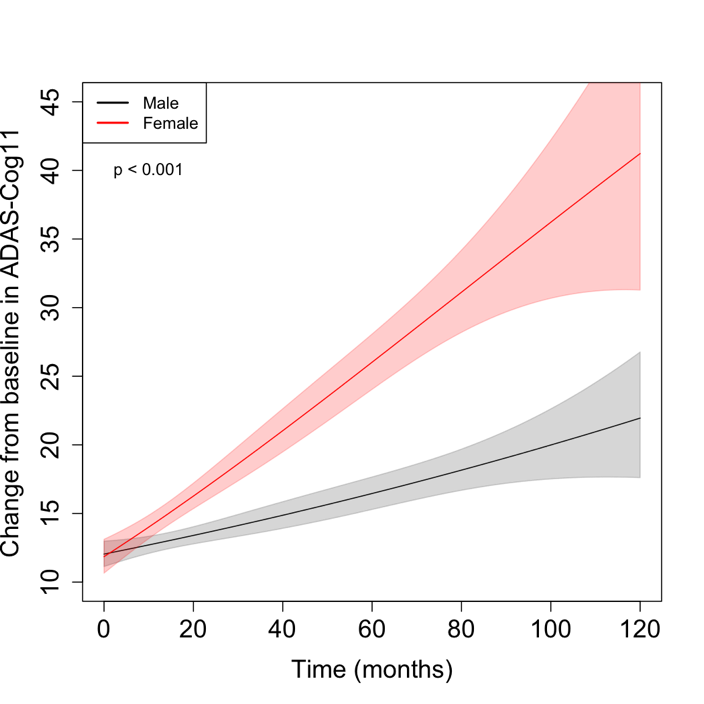

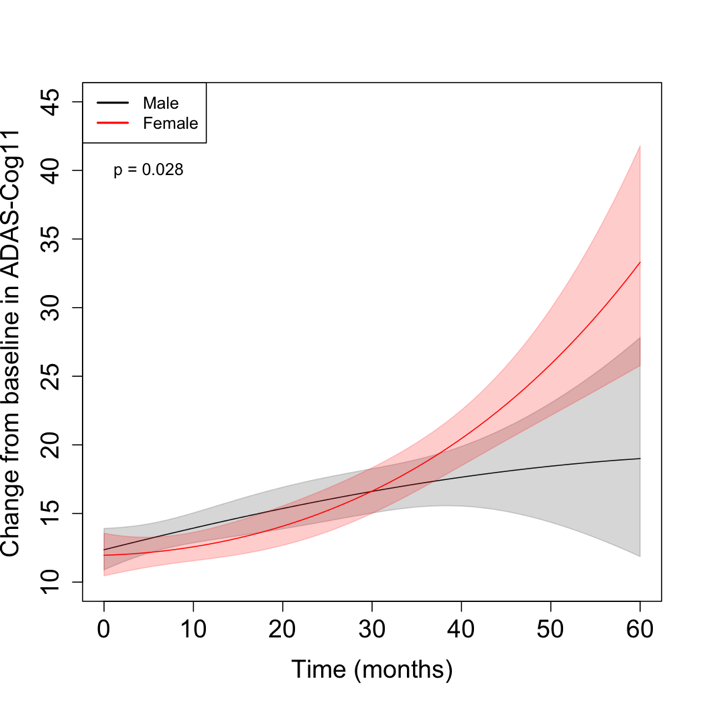


Figure depicts slopes of ADAS-Cog11 by sex in subjects with MCI due to AD – high likelihood. The x-axis depicts maximum duration of follow up. The Y-axis depicts ADAScog11 scores and is identical for both panels. Slopes for males and females are derived from a simple quadratic model by sex over time without any other covariates. ADNI-1 subjects had longer follow up and hence greater change. ADNI-1 also had a much larger sample size than ADNI-2. The effect of sex is significant in ADNI-1 but not in ADNI-2.

Supplementary table 1. Effect of interaction between Sex and *APOE* ε4 on longitudinal change in ADAS-Cog11 of MCI subjects

| Term | Coefficient | Standard error | t-value | p-value |
| --- | --- | --- | --- | --- |
| Intercept | 3.562517 | 0.07590739 | 46.93242 | **<0.001** |
| Female | 0.207763 | 0.12484928 | 1.66411 | 0.100 |
| *APOE* ε4 + | 0.277468 | 0.11262743 | 2.46359 | **0.014** |
| *APOE* ε4 ++ | 0.527658 | 0.16766664 | 3.14707 | **0.002** |
| Baseline rate | 0.012771 | 0.00201982 | 6.32298 | **<0.001** |
| Baseline curvature | 0.000094 | 0.00001980 | 4.73720 | **<0.001** |
| Age | 0.011442 | 0.00566260 | 2.02059 | **0.044** |
| Education | 0.017313 | 0.01437100 | 1.20471 | 0.229 |
| Baseline cognition | 0.166186 | 0.00939241 | 17.69364 | **<0.001** |
| Female * *APOE* ε4 + | 0.083238 | 0.18119540 | 0.45938 | 0.646 |
| Female * *APOE* ε4 ++ | 0.115644 | 0.26583082 | 0.43503 | 0.664 |
| Female effect on slope | 0.006720 | 0.00333667 | 2.01403 | **0.044** |
| Female effect on curvature | 0.000024 | 0.00003511 | 0.67896 | 0.497 |
| *APOE* ε4 + effect on slope | 0.010714 | 0.00301207 | 3.55701 | **<0.001** |
| *APOE* ε4 ++ effect on slope | 0.017095 | 0.00451578 | 3.78551 | **<0.001** |
| *APOE* ε4 + effect on curvature | 0.000092 | 0.00003136 | 2.93378 | **0.003** |
| *APOE* ε4 ++ effect on curvature | 0.000102 | 0.00004566 | 2.23384 | **0.026** |
| Education effect on slope | 0.000780 | 0.00038620 | 2.01947 | **0.044** |
| Age effect on slope | 0.000137 | 0.00015275 | 0.89908 | 0.369 |
| Baseline cognition effect on slope | 0.001076 | 0.00025591 | 4.20334 | **<0.001** |
| Female * *APOE* ε4 + on slope | -0.001397 | 0.00490124 | -0.28510 | 0.776 |
| Female * *APOE* ε4 ++ on slope | 0.006584 | 0.00747048 | 0.88133 | 0.378 |
| Female * *APOE* ε4 + on curvature | -0.000067 | 0.00005573 | -1.21034 | 0.226 |
| Female * *APOE* ε4 ++ on curvature | 0.000088 | 0.00009686 | 0.91029 | 0.363 |

Baseline cognition indicates ADAS-Cog 11. Bold *p*-values are statistically significant. Abbreviations: MCI (mild cognitive impairment), ADAS-Cog11 (Alzheimer’s disease assessment scale- cognitive subscale), and *APOE* ε4 (apolipoprotein ε4 allele). In this model, the follow-up time (month) was centered with the median follow-up time (36 months) and covariates were centered i.e. a 75 years old *APOE* ε4- male with 16 years of education and an ADAS-Cog11 of 11. Both sex and APOE ε4 had significant effects on ADAS-Cog11 slope but the interaction effect between sex and APOE ε4 was not significant. The intercept is a term to get the correct estimate of the outcome when time = 0.  The baseline rate is the reference population rate of change in the outcome starting at time zero, and the baseline curvature is the "acceleration" of that rate of change at time zero.  The upper half of the table shows the effect of specific variables on ADAS-Cog11 and the bottom half shows their effects on ADAS-Cog11 slope and curvature.

Supplementary table 2. Effect of Sex and CSF t-tau on longitudinal change in ADAS-Cog11 of MCI subjects

| Term | Coefficient | Standard error | t-value | p-value |
| --- | --- | --- | --- | --- |
| Intercept | 3.675458 | 0.06717570 | 54.71410 | **<0.001** |
| Female | 0.163752 | 0.11005364 | 1.48793 | 0.138 |
| Baseline t-tau | 0.006524 | 0.00100040 | 6.52184 | **<0.001** |
| Baseline rate | 0.017779 | 0.00182986 | 9.71584 | 0.256 |
| Baseline curvature | 0.000152 | 0.00002000 | 7.61322 | **0.002** |
| Age | 0.002261 | 0.00692640 | 0.32649 | 0.744 |
| Education | 0.008685 | 0.01841669 | 0.47157 | 0.638 |
| Baseline cognition | 0.145145 | 0.01149431 | 12.62756 | **<0.001** |
| Female effect on slope | 0.004479 | 0.00306824 | 1.45988 | 0.145 |
| Female effect on curvature | -0.000023 | 0.00003671 | -0.63791 | 0.524 |
| Baseline t-tau effect on slope | 0.000168 | 0.00002883 | 5.82222 | **<0.001** |
| Baseline t-tau effect on curvature | 0.0000003 | 0.00000038 | 0.91397 | 0.361 |
| Education effect on slope | 0.000547 | 0.00050227 | 1.08895 | 0.276 |
| Age effect on slope | -0.000058 | 0.00019012 | -0.30333 | 0.762 |
| Baseline cognition effect on slope | 0.000589 | 0.00031757 | 1.85432 | 0.064 |

Baseline cognition indicates ADAS-Cog 11. Bold *p*-values are statistically significant. Abbreviations: MCI (mild cognitive impairment), ADAS-Cog11 (Alzheimer’s disease assessment scale- cognitive subscale), *APOE* ε4 (apolipoprotein ε4 allele), and CSF (cerebrospinal fluid). In this model, the follow-up time (month) was centered with the median follow-up time (36 months) and covariates were centered i.e. a 75 years old *APOE* ε4- male with 16 years of education, an ADAS-Cog11 of 11, and CSF t-tau of 84.5. In this model the effect of t-tau on ADAS-Cog11 slope was significant but the effect of sex was not. The baseline rate is the reference population rate of change in the outcome starting at time zero, and the baseline curvature is the "acceleration" of that rate of change at time zero.  The upper half of the table shows the effect of specific variables on ADAS-Cog11 and the bottom half shows their effects on ADAS-Cog11 slope and curvature.

Supplementary table 3. Effects of Sex and Hippocampal Volume on ADAS-Cog11 change in MCI subjects

| Term | Coefficient | Standard error | t-value | p-value |
| --- | --- | --- | --- | --- |
| Intercept | 3.827073 | 0.05615707 | 68.14945 | **<0.001** |
| Female | 0.177931 | 0.09391647 | 1.89457 | 0.059 |
| Hippocampal volume | -0.000293 | 0.00004785 | -6.11468 | **<0.001** |
| Baseline rate | 0.021160 | 0.00151433 | 13.97300 | **<0.001** |
| Baseline curvature | 0.000136 | 0.00001606 | 8.47927 | **<0.001** |
| Age | -0.015311 | 0.00669038 | -2.28851 | **0.023** |
| Education | 0.017512 | 0.01491466 | 1.17412 | 0.241 |
| Baseline cognition | 0.155126 | 0.01042223 | 14.88415 | **<0.001** |
| Female effect on slope | 0.004542 | 0.00255732 | 1.77595 | 0.076 |
| Female effect on curvature | -0.000005 | 0.00002729 | -0.19099 | 0.849 |
| Hippocampal volume effect on slope | -0.000007 | 0.00000130 | -5.64763 | **<0.001** |
| Hippocampal volume effect on curvature | 0.000000 | 0.00000001 | -0.12413 | 0.901 |
| Education effect on slope | 0.000851 | 0.00040511 | 2.09960 | **0.036** |
| Age effect on slope | -0.000568 | 0.00018286 | -3.10350 | **0.002** |
| Baseline cognition effect on slope | 0.000833 | 0.00028581 | 2.91421 | **0.004** |

Total hippocampal volume (left plus right) was extracted from baseline MRI using Freesurfer. Baseline cognition indicates ADAS-Cog 11. Bold *p*-values are statistically significant. In this model, the follow-up time (month) was centered with the median follow-up time (36 months) and covariates were centered i.e. a 75 years old with 16 years of education, an ADAS-Cog11 of 11, total hippocampal volume of 5861. The effect of total hippocampal volume on ADAS-Cog11 slope was significant but the effect of sex was not. The baseline rate is the reference population rate of change in the outcome starting at time zero, and the baseline curvature is the "acceleration" of that rate of change at time zero.  The upper half of the table shows the effect of specific variables on ADAS-Cog11 and the bottom half shows their effects on ADAS-Cog11 slope and curvature.
